# Engaging East Harlem, New York youth in action gun violence prevention research and child rights: a preliminary study

**DOI:** 10.1186/s40621-023-00471-4

**Published:** 2023-11-21

**Authors:** Pallavi Malla, Nakesha Fray, Margaret K. Formica, Danielle Goldberg, Robert Marchesani, Patricia Hennessy, Moshay Ervine, Jacqueline G. Wallace, Elaine Larson, Pamela Wridt, Danielle Laraque-Arena, IAACT, Adam Mrozowski, Alexander Troung, Alina Lugo, Benjamin Hoffman, Brianna Haughton, Dibrianys Duran, Danielle Goldberg, Danielle Laraque-Arena, David Kener, Dyanand Sugrim, Elaine L. Larson, Jacqueline G. Wallace, Jasmin Hari, Kimber Bogard, Margaret Formica, Mariela Reyes, Michael Canfora, Mohammad Shajee, Moshay Ervine, Nakesha Fray, Nina Agrawal, Pallavi Malla, Pamela Wridt, Patricia Hennessy, Paul Theerman, Reggie Richards-Peelle, Robert Marchesani, Roseanne L. Flores, Shaneah Taylor, Syanne Castro, Yvonne Graham

**Affiliations:** 1 Mailman School of Public Health, Columbia University, New York, USA; 2 Department of Public Health and Preventive Medicine, SUNY Upstate Medical University, New York, NY USA; 3 UNICEF, New York, NY USA; 4 Counseling in Schools (CIS), New York, NY USA; 5 The Heritage School, East Harlem, New York, NY USA; 6 Icahn School of Medicine at Mount Sinai, New York, NY USA; 7 New York Academy of Medicine (NYAM), 1216 Fifth Avenue, New York, NY 10029 USA; 8 Conscious Data Inc, Brooklyn, New York, NY USA; 9 Intergenerational Action Adolescent & Child Team, a collaboration of the NYAM, Columbia University, Mailman School of Public Health Department of Epidemiology, UNICEF USA, Counseling in Schools and The Heritage School, New York, NY USA

**Keywords:** Child rights, Gun violence, Youth, Community safety, YPAR, Participatory action research, CFCI

## Abstract

**Background:**

The aim of the study was to have youth participate in the design and implementation of a research project set within a child rights framework to better understand high schoolers’ perceptions of safety in their school and community.

**Results:**

Between June 2020 and March 2021, a team of East Harlem, New York high school students, participated as co-researchers to modify the United Nations Children's Fund Child Friendly Cities Initiative Survey to suit their needs. Due to the COVID-19 pandemic, the final survey was conducted through an online remote classes system during advisory school classes, accompanied by brief focused group discussions. The novel process of conducting an interactive qualitative and quantitative virtual survey during a pandemic via youth participatory action research is outlined in this paper.

**Conclusions:**

Our results demonstrate that youth participatory action research can be utilized as part of a child rights framework approach to assess the views of youth regarding community safety and violence prevention.

## Introduction

Research incorporating youth identified priorities is urgently needed to develop appropriate and sustainable interventions to improve youth’s sense of feeling safe in their community. The Child Rights Framework (CRF) is based on the premise that all youth, without discrimination, must have the opportunity to represent themselves, tell their stories, and gain access to vital information to promote positive social change to their environment (UNICEF’s Child Rights Framework 2023). The CRF can help elucidate larger systemic issues that may be affecting youth and get to the root of the problems (UNICEF’s Child Rights Framework 2023; Ozer 2016; Laraque et al. 1994, 1995).

Youth-led participatory action research (YPAR) engages young people in conducting research to identify problems in their lives as well as advocate for changes, while also providing insider expertise on their experiences. YPAR engages young people to serve as research collaborators, helps youth be the agents of positive change in their community, provides opportunities for youth to see themselves as leaders (Prati et al. 2020), and decreases scores of political alienations (Gibbs et al. 2020).

The COVID-19 pandemic placed a heavy burden on the public health system, and youth, as a vulnerable population, were deemed at risk for the mental health effects imposed by the pandemic such as social isolation and loss of the usual community, school and peer supports (Altheimer et al. 2020). The psychosocial ill-effects of the pandemic have been shown to compound children and adolescents’ behavioral response (Singh et al. 2020). For these reasons, now more than ever, YPAR, in which young people can express their views and claim their rights, is important.

The current study was sponsored by the New York Academy of Medicine (NYAM) in response to local concerns regarding youth safety and the gun violence (GV) epidemic. A student at The Heritage School lost their life to a gun violence related incident. The Heritage School was chosen to pilot this program because of the history of gun violence in East Harlem, the interest that the school had taken with regards to gun violence, as well as the proximity of The Heritage School to NYAM. With this perspective in mind, the New York Academy of Medicine partnered with UNICEF USA and Counseling In Schools and used UNICEF’s Child Friendly Cities Initiative’s (CFCI) child rights framework, to integrate youth as co-creators and collaborators in a project to understand youth perceptions of factors that affect their physical, social, and psychological well-being in their schools and communities. The aims of this project were to (a) involve high school youth as partners to modify the UNICEF Child Friendly Cities Initiative Survey to reflect their perceptions and the environment and (b) use the survey to examine adolescent’s perceptions of safety in their school and community as it relates to children’s rights and GV. By collaborating directly with youth, we hoped to identify salient points to guide research useful in targeted interventions.

This study was reviewed and approved by the New York Academy of Medicine Institutional Review Board.

## Methodology

To design and implement the project, an Intergenerational Action Adolescent and Child Team (IAACT) was formed consisting of a collaborative group of local and global organizations. In this project, an informal group of students from The Heritage School initially met with all the collaborators to express their safety concerns and priorities and helped define the aims of the project and its focus on GV. They subsequently selected among themselves students representing several grades/ages, to participate in the design of the study, pilot testing and to serve as liaisons for the project. Constant dialogue was maintained by involving all the stakeholders in biweekly meetings. The stakeholders’ roles were to provide administrative support, resources, review of survey instruments, research design including human subject considerations, and guidance for the successful implementation of the project virtually.

Youth actively participated, initially in person and, during COVID restrictions, via remote media in all phases of the project including development and testing of survey content, research design, implementation, data collection, and analysis. They utilized graphical tools and multimedia software to design the study instrument, created online discussion groups, and facilitated discussions through online video platforms. They engaged in conference calls with the sponsors and stakeholders and worked with school administrative authorities to plan and conduct the research.

### Survey development

The initial survey instrument considered for this study was the global Child Friendly Cities Initiative (CFCI) Community Assessment Survey as adapted by UNICEF USA for the United States context (Child Friendly Community Self-Assessment Tool for Adolescents 2023; Children’s Environments Research Group (CERG) 2014; Wridt 2015; Wridt et al. 2015). The heart of the toolkit is the CFCI community assessment survey for different age groups, which can be facilitated by adolescents in partnership with adult allies. The surveys are implemented using local objects, stickers, and through play-based approaches that are developmentally appropriate for children of different ages and abilities. The survey uses simply worded, developmentally appropriate statements on community conditions that are linked to specific articles from the Convention on the Rights of the Child (Wridt 2015). In this study, we followed the recommended procedures for adapting the CFCI survey, which involves forming a committee of interested adults and youth to review and modify the survey statements and images for their local context. Five student interns, through facilitated weekly discussions with IAACT members, reviewed 64 survey statements for youth ages 13–18 years from the CFCI interactive tool, 34 of which were deemed to be core questions. The survey was categorized in 5 domains: ‘My Safety and Inclusion,’ ‘My Participation,’ ‘My Play and Leisure,’ ‘My Community Service,’ and ‘My Living Environment.’ The discussions were guided by two public health graduate students (Malla, Fray), and the group met weekly over an 8-week period to review and modify the CFCI assessment survey. Biweekly meetings were conducted with stakeholders at their convenience to discuss the youth modifications to the survey and provide general edits. The youth found most of the required survey items as relevant for EH, but also reported that optional questions pertaining to public transportation, sexual harassment, drug dealing, and illegal activities were important to the urban context in which they lived, so these statements were included. In addition to selecting the most relevant survey's statements, the IAACT members made some of the statements more specific to EH and their school and considered ways of adapting the images for EH. In the end, the IAACT members did not have the necessary skills to adapt the images for EH, and opted to remove them since the population was older, literate youth.

The second component of the survey considered for this study was related to GV. Validated questions were adapted from the Center for Disease Control and Prevention (CDC) Youth Risk Behavior and Surveillance System (YRBSS) questionnaires for high school students relating to safety, violence, weapon use, and mental health during COVID-19 (Youth Risk Behavior Survey Questionnaire 2019). The students felt the need to address GV specifically since the CFCI did not directly query regarding guns. The school had one of its own students killed by GV, engaged students from Marjory Stoneman Douglass high school in Parkland Florida where a mass shooting had taken the lives of 17 on February 14, 2018, and felt the need to add gun-related questions to the survey. In addition, given the context of the pandemic, the students indicated the need for questions related to the pandemic’s impact on their families and their mental health. Thus, six questions from the YRBSS were added to the complement of the CFCI questions chosen by the students.

The IAACT team followed the recommendation that adolescents be involved in facilitating the surveys. Prior to this study, there were no known cases of facilitating the survey administration online. Staying true to the interactive, real-time data analysis and discussion-based approach for facilitating the survey process, the IAACT team selected PollEverywhere® as its tool. Students and the team were trained in survey implementation using the PollEverywhere® platform. Students also participated in the design of focused group discussions which were to follow the administration of the survey to clarify both content and wording of the questions as well as other topic areas of interest.

The survey was initially pilot tested with 8 students. Following the pilot survey, Carman et al. (2016) questions chosen by the students and team were discussed in an open-ended fashion. These questions included topics on feeling safe, sexual harassment in public spaces, and drug dealing in the community. Discussions also led to the addition of an open-ended question to describe concerns not addressed in the survey. Additional questions on GV and COVID-19 taken from the CDC Youth Risk Behavior Survey for high school students and questions on stress and sexual harassment were added (Youth Risk Behavior Survey Questionnaire) after discussions led by students (Youth Risk Behavior Survey Questionnaire 2019). The final survey consisted of 44 CFCI questions, six YRBSS questions, five demographic questions, one question on GV, and one open-ended question.

### Study population

All students, grades 9–12 of a high school in EH were eligible to participate.

The total student population was 66% Latinx, 27% Black, 3% White, 2% Asian, and 2% other races/ethnicities. The school reported 70–74% graduation rate and 80 to 89% of students were proficient in math and more than 90% were proficient in reading. Eighty-eight percent of students were eligible for free lunch, and 4% of students were eligible for reduced lunch.

### Survey implementation

The youth community assessment was conducted via Zoom® during school advisory class periods, which were recently mandatory during the 2020–21 academic year. Total enrollment for the school was 304 students with an average attendance of about a quarter of the students. The survey was administered over two days in 14 different advisory sessions in March 2021 while students were still learning remotely.

Sessions were moderated by classroom teachers, counselors who served to introduce the session, and logistics moderators who navigated the technical aspect of the survey administration and monitored the chat function during the focused group discussion. At the start of each class period, all students could enter the Zoom session in audio mode only and the logistic moderator instructed the students to change their name for confidentiality reasons to their favorite cartoon character followed by 2 numeric digits. Logistics moderators assisted with name changes, introduced the survey and PollEverywhere® instructions, provided clarifications, and shared response summaries on Zoom for the focused group discussions. In addition, logistic moderators ensured that all student cameras remained turned off. The teachers and Counseling In Schools counselors known to the students served as discussants.

### Analytical methods

Univariate frequencies and proportions were obtained for each question on the scale (mostly true, sometimes true, never true, I don’t know). To evaluate whether responses differed by demographic characteristics, bivariate analyses were performed examining the distribution of the responses by self-identified gender, race, and area of residence. Because of the limited sample size of the study, the data were sparsely distributed when analyzed as ordinal categorical values. To better interpret the data, categories were collapsed into mostly true versus others to obtain two-by-two contingency tables. Chi-square statistic was used to test if the two variables were associated, and relative risks (RR) with corresponding 95% confidence intervals were computed to quantify the magnitude of the association. Fisher’s exact test was performed when the expected cell frequency was less than 5. The level of youth participation was analyzed retrospectively using Shier’s analytical tool (Shier 2018).

## Results

### Survey participation

Table 1 demonstrates the final survey statements. Students made modifications to clarify wording and added questions guided by the examination of the fuller compendium of questions from the CFCI interactive tool.

**Table 1:** Final survey statements developed by the students and utilized within the youth community assessment pilot

Topic/Indicator	Convention on the Rights of the Child (CRC) Articles	Original Survey Statement	Modified Survey Statement after Input from Students^a^
Community Safety	6, 19, 34, 35, 36, 37, 38, 39	I feel safe in my community (protected from abuse, gangs, etc.)	I feel safe in my community (protected from abuse, gangs, etc.)
Gender Equality	2, 12, 13	Girls and boys are treated equally in my community	All genders are treated equally and given the same opportunities
Protection from Bullying	6, 19, 27, 34, 35, 36, 37, 38, 39	I feel safe from being bullied by other youth at school and/or online	I feel safe from being bullied by other children at school and/or online
School safety	19, 28, 36	I feel safe at school	I feel safe socially/physically/emotionally at school
Inclusion of Children with Disabilities	23, 28, 29	Children with disabilities are respected and given equal treatment in my community	Children with mental health needs and physical disabilities are respected and given the same opportunities
Diverse Friendships	2, 12, 13, 14, 30	I have friends of different origins, backgrounds, genders or abilities	I have friends of different origins, backgrounds, genders or abilities
Police-Community Relations	6, 24, 27	I trust police officers and other security guards in my community	I trust police officers and feel they are allies in my community
Protection from Gangs	19, 34, 35, 38	I feel protected from gangs or armed groups in my community	I feel protected from gangs or armed groups in my community
Social Support	6, 18, 19, 27, 34, 35, 36, 37, 38, 39	There are adults in my community who I can talk to freely about abuse or violence	There are adults in my community who I can talk to freely about abuse or violence

^a^Final questions used in the survey after discussions and feedback from students. Survey participants had to choose from one of the following responses: Mostly True; Sometimes True; Never True, I do not know

The survey was conducted during the lockdown period of the COVID pandemic. As a result, of the 304 enrolled students, a total of only 64 eligible participants were present virtually in their classes. Of these, 63 were willing to participate in the study. Nine students were unable to participate due to technical or connectivity issues, resulting in a total of 54 participants. Seven students did not provide basic demographic information and 7–9 (13–17%) students chose not to answer various questions relating to GV or violent attacks. In testing this modified survey, we noted that questions relating to the domain of ‘My Safety and Inclusion’ had the most responses with only 2 to 3 students choosing not to answer these questions.

A total of 30 students participated in focused group discussions. Focused group discussions were facilitated by proctors and conducted over the 14 advisory sessions. The mixed method mirrored the global experience of UNICEF of discussing directly with youth their ideas and clarification of survey elements. Nuanced responses and complexities were better understood using a mixed method approach which allowed students to raise issues that they did not see emphasized in the survey and to highlight contextual issues relevant to the experience of living in their particular community.

The approach of involving youth in the planning, designing, and implementation of the research study demonstrated a high level of participation by youth as assessed retrospectively within the categories outlined by Shier (2018).

### Salient survey results

Of the 54 participants, for those responding to the question on gender, 18 (38%) were male, and 26 (55%) were females. Three students (6%) preferred not to disclose their gender. When responding regarding race/ethnicity, most of the participants (68%) classified themselves as Latinx. Though the high school is in EH, 55% reported not living in EH.

In the domain of ‘My Participation’ and ability to influence decisions, 80% (40/50) were aware that children have rights and 78% (40/51) stated that it was mostly or sometimes true that they have opportunities to voice their opinions or concerns on decisions that affect them in their school. In contrast, only 35% (17/48) expressed that they were always or sometimes involved in planning or decision-making in the community. A large proportion, 92% (46/50) of the students, always or sometimes reported trying to learn about political issues that affect young people in their community.

In the domains of ‘My Community Service’ and ‘My Living Environment’ over 90% (44/47) felt safe at home and that the air in the house was clean and free of dust, toxins, and mold. More than 80% percent felt that they could often access care when they were sick or for regular health checkups (40/48) and felt comfortable when they went for health checkups (39/48). All students responded that to some extent they have enough nutritious food to eat at home or school every day.

When responses were compared across gender, females were more likely to report feeling vulnerable. On the question of feeling safe from being bullied by other children in school or online, 48% (12/25) of females reported feeling safe most of the time compared to 76% (13/17) of males (*p* = 0.07). Compared to females, males felt significantly safer from being sexually harassed, 72% (13/18) vs. 31% (8/26), (*p* = 0.007) and significantly safer to walk or ride a bike in the community, 61% (11/18) vs. 23% (6/26) (*p* = 0.01). More males than females reported that there were spaces for play and leisure near their homes that they use (e.g., parks, basketball courts, etc.), 83% (15/18) vs. 50% (13/26) (*p* = 0.02).

Since the number of students identifying as non-Latinx were sparsely distributed, analysis groups were categorized as Latinx background or other. Fewer students identifying themselves as Latinx reported feeling that security guards kept their community safe compared to others, 17% (5/29) vs. 53% (8/15), (*p* = 0.01). Similarly, compared to Latinx, non-Latinx felt protected against gangs and armed groups 60% (9/15) vs. 28% (8/29), (*p* = 0.03).

None of the students witnessing GV felt that they trusted police officers to keep them safe (0/12) compared to 38% (12/32) among those who never witnessed GV (*p* = 0.01). Compared to students who did not witness gun violence, students who witnessed GV were 2.4 times more likely to learn about political issues that affect young people in their community 8/12 (67%) versus 9/32 (28%), RR = 2.37 (95%CI 1.20–4.69, *p* = 0.02).

Students not witnessing a physical attack in their neighborhood were more than 3 times more likely to report feeling safe and protected in their community (17/27) compared to those who witnessed assaults in their neighborhood (3/16), RR = 3.36 (95% CI 1.16–9.69; *p* = 0.005) and protected from gangs and armed groups (14/27) vs. (3/17), RR = 2.93 (95% CI 1.00–8.73; *p* = 0.03). Compared to students who have seen a violent crime incident, students not witnessing violent crime were 2.7 times more likely to report that there were adults in their community whom they could talk to freely about abuse and violence (13/27) compared to those who had seen attacks (3/17), RR = 2.73 (95% CI: 0.91–8.19; *p* = 0.06).

### Focused group discussion results

In the subset of students who participated in the focused group discussion through Zoom®, many students reported feeling safe at home and at school, but unsafe walking or going to school, or even stepping foot into their apartment complex or going to the local bodega. Female youth felt that sexual harassment was a major problem outside and referenced “old creepy men” that would hang around the school and catcall them. One individual stated that despite police presence in the area, sexual harassment was still very prevalent, and that change had to come from education at a young age. Also discussed were questions related to drug dealing, gangs, and illegal activity. Students discussed that they were not blind to drug dealing and that illegal activity was inevitable in their neighborhoods. They mentioned a pervasive “smell in the air” of drugs. Finally, COVID-19 and mental health was a major area of concern for students. Of the individuals who responded to the open-ended question on topics that were important to them, 7/30 (23%) cited COVID-19 and mental health to be an area they viewed as important. There was a general feeling of fatigue regarding the pandemic and community safety. However, some students expressed feelings of hope that they could affect positive change with their voices.

## Discussion

A YPAR approach in the examination of GV and community safety within the framework of child rights in the USA using a virtual platform is novel and may elucidate several areas for development of survey methodology and for possible avenues to gain the views of youth on interventions to reduce both the experience with GV, sexual harassment and promote youth well-being. The use of YPAR in the local context of EH, a predominantly Latinx, underserved community, provides a framework for understanding youth acceptance of this approach and understanding the youth voice and planning for public deliberation on how this knowledge could be used to propose solutions to the problems identified by youth (Carman et al. 2016). As noted by a number of researchers, addressing salient health areas such as substance use, obesity, food insecurity, mental health, social capital, sexuality, as well as community safety and violence, methods for engagement of youth in their community context is important in developing interventions that respond specifically to youth concerns in specified neighborhoods. This approach can be replicated, and youth participation can lend credence to this approach.

Various tools to evaluate the level of youth participation in a study have been published including Hart’s Ladder of Participation, Minkler’s reliability-tested guidelines for assessing participatory research projects, and Shier’s analytical tool (Shier 2018; Hart 1992; Minkler and Wallerstein 2011). As noted, our processes demonstrated a high level of participation by youth who attended class and can serve as a model for replicating youth involvement as co-researchers. The questions most relevant to the students, paraphrased in the way they and their peers would understand them, the social context, and the description of the environmental conditions germane to them, were what they decided to include in the survey. Expanding this survey method to other neighborhoods in this and other city schools may help determine if the domains and topic issues raised by the EH students translate across different neighborhoods and what relevant adaptations may be needed to respond to local context.

As a preliminary study, there were some salient results that can be explored with larger samples. For example, a quarter of the students expressed mistrust of police, suggesting that school-based interventions may be necessary for police to gain the trust of youth. Notably, females perceived greater threats to being bullied and sexually harassed. This finding may indicate the need to better understand perceptions of safety that differ by gender. Additionally, efforts could be made by having youth speak directly to authorities to elucidate underlying reasons behind underutilization of existing facilities, as well as, urging the development of safe, welcoming public space especially designed to meet the needs and interests of female students, utilizing a youth participatory project for urban planning. Such placed-based interventions have resulted in positive outcomes including decreases in injury rates (Durkin et al. 1996; Hohl et al. 2019; Zinzow et al. 2009).

Our data suggest that students who have witnessed gun-violence or violent crime are threatened by their environment and do not feel safe in their community. In this small sample, students reported being less likely to reach out to adults in the community for help or discuss problems about abuse and violence. These individuals could be at risk of subsequent mental health problems and future studies can explore this possibility (Zinzow et al. 2009; Turner et al. 2019).

Our results indicate that youth can be co-researchers, recognize their rights, and can bring attention to issues previously neglected. The issue of sexual harassment of female students did emerge as an issue which was not the focus of the study, but that students brought to our attention.

This preliminary study was able to demonstrate the feasibility of engaging youth in the design and implementation of a research project focused on GV, community safety, and child rights. Too often, programs are developed without considering the thoughts and direct input of the youth who are affected (Bowen et al. 1998).

## Conclusion

Our results demonstrate that youth participatory action research can be utilized as part of a child rights framework approach to assess the views of youth regarding community safety and violence prevention. It is feasible to involve youth as co-researchers in the design and implementation of projects on matters affecting them. Interventions are needed to ensure that youth feel safe, with emphasis on gender-based harassment.

## Data Availability

The datasets used and/or analyzed during the current study are available from the corresponding author on reasonable request.

## Abbreviations

CDC: Centers for Disease Control and Prevention
CERG: Children’s Environmental Research Group
CFCI: Child Friendly Cities Initiative
CRC: Convention on the Rights of the Child
CRF: Child Rights Framework
EH: East Harlem
GV: Gun violence
IAACT: Intergenerational Action Adolescent & Child Team
UNICEF: United Nations Children's Fund
YPAR: Youth Participatory Action Research
YRBSS: Youth Risk Behavior Surveillance Survey
